# 

**Published:** 1903-03-15

**Authors:** 


### ANNOUNCEMENTS.

THE Louisville Dental College Alumni Association will hold its annual meeting in the College, on Friday and Saturday, March 27th and 28th. All Alumni and the profession generally are cordially invited to attend. An interesting program will be provided.

DR. T. W. BROPHY and Dr. A. W. Harlan will represent the United States at the International Medical Conference, to be held in Madrid, Spain, in April.

THE Northwestern University has conferred upon Dr. E. C. Kirk the honorary degree of Doctor of Science. A meritorious compliment.

A COMPLIMENTARY dinner was given in New York, January 21st, to celebrate the fifty-fifth year of continuous practice of the dental profession by Dr. C. S. Stockton, of Newark, N. J. That is a long time to spend in any business and certainly attests the physical strength as well as the affection for his profession which are characteristic. Dr. Stockton has always taken a great interest in the public welfare of his profession and has a wide acquaintance.

DR. WM. C. BARRETT, of Buffalo, met with a bad accident which has laid him up temporarily. He slipped on the icy sidewalk and fell, dislocating his shoulder. It is hoped that he may make a speedy recovery in spite of the serious nature of the injury.

—✦—

THE officers elected by the Institute of Dental Pedagogics for the year are: J. D. Paterson, Pres; H. B. Tileston, Vice Pres.; W. Earl Wilmott, Secretary-Treasurer; Executive Committee, W. H. Whitslar, D. R. Stubblefield and R. H. Nones. The next meeting will be held at Buffalo, in December.

—✦—

THE Central Michigan Dental Association will hold its next meeting at Grand Ledge, May 13th and 14th. An interesting program is in preparation, and a banquet with special features. A cordial invitation is extended to all interested.

—✦—

THE Southwestern Michigan Dental Association will hold its semi-annual meeting at Albion, April 7th and 8th. President Warboys will be the host and he assures all of a cordial welcome, and promises an attractive program, and a good time. This is one of the societies where a good social time is always an important event.

